# Strengthening Nonspecialist Health Care Providers’ Capacity to Address Mental Health in the Context of Domestic Violence in Nepal: Pre–Post Mixed Methods Training Evaluation

**DOI:** 10.2196/72793

**Published:** 2026-02-12

**Authors:** Reena Koju, Rachana Shrestha, Jayanti Dhungana, Achyut Lamichhane, Diksha Sapkota, Anna Mia Ekström, Keshab Deuba

**Affiliations:** 1 Public Health and Environment Research Centre (PERC), Nepal, Sanepa, Lalitpur, Nepal; 2 Knowledge to Action (K2A), Lalitpur, Nepal; 3 Department of Global Public Health, Karolinska Institutet, Norrbackagatan 4, Solna, Stockholm, 17176, Sweden, 977 9843064279 ext 01; 4 Department of Psychiatry, Nepal Police Hospital, Kathmandu, Nepal; 5 Griffith Criminology Institute, Griffith University, Queensland, Australia; 6 Department of Infectious Diseases/Venhälsan, Södersjukhuset, Stockholm, Sweden; 7 Global Health Epidemiology Group, Department of Global Public Health and Primary Care, Centre for International Health (CIH), Bergen, Norway

**Keywords:** health care providers, mental health training, knowledge and attitudes, safety planning, Problem Management Plus, Nepal, training effectiveness

## Abstract

**Background:**

Health care providers (HCPs) in public health facilities in low- and middle-income countries, including Nepal, often lack adequate training to manage mental health problems effectively.

**Objective:**

This study evaluated the impact of structured mental health training on the knowledge, attitudes, confidence, and psychosocial support skills of nonspecialist HCPs in Madhesh Province, Nepal.

**Methods:**

This study is a nested substudy within a larger domestic violence (DV) intervention trial and used a mixed method, pre–post intervention design with a comparison group. A total of 46 nonspecialist HCPs were randomized into 2 groups: group 1 (n=24) received a 10-day comprehensive mental health and violence prevention training; group 2 (n=22) received a 3-day training focused on ethical considerations, the link between intimate partner violence (IPV) or DV and mental health, and available referral services. The training was based on the World Health Organization’s Problem Management Plus model, with augmented modules on safety planning and psychosocial support. Changes in knowledge and attitude scores were assessed at baseline, immediately post-training, and at 3-month follow-up. In-depth interviews with participants from group 1 were thematically analyzed.

**Results:**

At baseline, nearly 90% of nonspecialist HCPs had not received any prior formal mental health training. Both groups demonstrated significant improvements in mental health knowledge, with a greater increase observed in group 1 (mean score 41.33-48.41) compared to group 2 (41.18-44.27). Attitudes toward individuals with mental health problems also improved in both groups, reflected in reductions in social distance and perceived dangerousness scores. Thematic analysis of interviews indicated enhanced confidence and psychosocial support skills, particularly in managing mental health concerns among women experiencing IPV or DV.

**Conclusions:**

Structured mental health training significantly improved both knowledge and attitudes among nonspecialist HCPs in public health facilities in Madhesh Province. Participants also reported increased confidence in addressing common mental health concerns. This training model has potential for scale-up in other resource-limited settings to build frontline capacity in managing mental health problems and supporting women experiencing IPV or DV.

## Introduction

Globally, an estimated 1 in 8 individuals experience mental health problems, particularly anxiety and depression [1]. The burden of these conditions increased substantially during the COVID-19 pandemic [2], driven by factors such as job losses, social isolation, and mobility restrictions. These disruptions also contributed to a rise in violence against women worldwide [3]. Despite this growing burden, access to mental health services remains limited in many settings, largely due to a global shortage of trained mental health professionals [4].

Mental and physical health are closely interconnected. Individuals with mental health problems face a higher risk of developing other health problems, including HIV, tuberculosis, and noncommunicable diseases [1]. Left untreated, mental health conditions can lead to long-term disability, unemployment, substance use, and even suicide [5], which is a leading cause of death among young people [1]. Mental health problems also contribute significantly to economic losses and societal burden [6]. In many low- and middle-income countries (LMICs), mental health remains a neglected area of public health, characterized by underfunding, limited service coverage, and substantial unmet needs [7,8]. Stigma and discrimination further hinder access to care [9].

In Nepal, as in many LMICs, mental health services are underresourced [10]. Government expenditure on mental health remains below 2% of the national health budget [11], and services are largely concentrated in tertiary care settings [12]. Although Nepal’s National Mental Health Strategy and Action Plan [13] advocates for the availability of psychotropic medications—such as antipsychotics, antidepressants, anxiolytics, mood stabilizers, and antiepileptics—at all health facility levels, chronic supply chain issues [14,15] and limited authority to dispense these medications at lower-tier facilities persist [14].

The mental health workforce in Nepal remains severely limited [16]. As of 2020, there were only 200 psychiatrists across the country [17]. Nepal has a single mental health hospital, 18 outpatient mental health care facilities, and 17 general or teaching hospitals with inpatient psychiatric units—all of which are based at the tertiary level [18]. With only 500 psychiatric beds nationally—approximately 1.5 beds per 100,000 people [19]—access to care is particularly limited in rural and remote areas. The 2021 Nepal Health Facility Survey found that only 25% of health facilities provided any mental health services, and this proportion was even lower outside urban centers [15]. Barriers such as stigma, poor awareness, discrimination, and logistical inaccessibility lead many people with mental health needs [20] to go untreated or to rely on informal providers, such as traditional healers [21].

To address the treatment gap, the World Health Organization’s (WHO) mental health Gap Action Program has been adopted in Nepal [22], training nonspecialist health care providers (HCPs) to identify and manage priority mental health conditions such as depression, psychosis, epilepsy, and substance use disorders [22]. Other initiatives, such as the REducing Stigma among HealthcAre ProvidErs (RESHAPE) program, have targeted stigma reduction through providers’ training in selected municipalities [23]. While these programs represent important progress, their implementation remains limited in scale, and they often do not address the complex mental health needs of women who have experienced intimate partner violence (IPV) or domestic violence (DV).

In Nepal, IPV is widespread. Nearly 29% of women aged 15 to 49 years reported experiencing emotional, physical, or sexual violence from an intimate partner in the previous 12 months [24]. Among those, 41% reported symptoms of anxiety, 33% reported depression, and 14% reported suicidal ideation. Yet, mental health utilization among women experiencing IPV remains low, with only 24% seeking support. Although HCPs are well positioned to identify and respond to mental health needs among IPV or DV survivors, evidence shows that most lack the necessary training and support [25]. The 2021 National Health Facility Survey found that only 27.1% of facilities offering mental health services had clinical guidelines in place, and just 16.2% of HCPs had received any form of mental health training prior to 24 months [15].

Evidence suggests that nonspecialist HCPs can play a pivotal role in early detection, psychosocial support, and referral for individuals with mental health problems, especially in low-resource settings [26,27]. Systematic reviews have shown that training nonspecialist providers in mental health care can improve their knowledge, confidence, and clinical practices, ultimately leading to better patient outcomes [26]. In Nepal, training programs targeting rural health providers have demonstrated a positive impact on managing depression and anxiety [28], with similar evidence reported from Ethiopia [27].

In response to the gap between rising mental health needs and shortage of trained professionals, particularly in underserved regions such as Madhesh Province, we implemented a structured mental health training program targeting nonspecialist HCPs. The training aimed to prepare frontline providers to identify and manage women experiencing IPV-related psychological distress. This study evaluates the impact of structured mental health training on the knowledge, attitudes, confidence, and psychosocial support skills of nonspecialist HCPs in Madhesh Province, Nepal. The findings contribute to ongoing efforts to integrate mental health and IPV/DV support into the public health system and may inform national strategies to build capacity among frontline health workers.

## Methods

### Study Design

This study employed a mixed methods design, combining quantitative and qualitative approaches to evaluate the impact of structured mental health training on nonspecialist HCPs. The quantitative component followed a pre- and post-test design with a comparison group to assess changes in mental health–related knowledge and attitudes. The qualitative component involved in-depth interviews with nonspecialist HCPs from group 1 (nonspecialist HCPs from the intervention sites of the parent cluster randomized trial, domestic violence intervention [DeVI]) to explore their experiences with the training and their perceived changes in knowledge, confidence, and psychosocial support skills.

### Setting

According to the Nepal Demographic and Health Survey, more than 1 in 5 women and 1 in 10 men aged 15 to 49 years report symptoms of anxiety [20]. In Madhesh Province of Nepal, approximately 27% of health facilities report offering some form of mental health services. However, only 35 of 246 health facilities have essential psychotropic medicines available, and most services are delivered through private providers [29]. Given these constraints, the training targeted nonspecialist HCPs working in government primary health care centers (PHCCs) and public hospitals across 8 districts of Madhesh Province. Although public hospitals are not part of the first-contact primary care tier, they remain central to the public health care system and routinely serve women seeking maternal and reproductive health care. Because the intervention focused on supporting women affected by IPV, including nonspecialist HCPs from public hospitals was considered appropriate.

This study was a nested substudy within the broader DeVI trial, a cluster randomized controlled trial designed to reduce IPV and related psychological distress among women. Details of the trial design are described elsewhere [30]. The training was conducted before the implementation of the main DeVI. A total of 24 public health facilities were randomly selected and assigned to either the intervention or control arms of the DeVI trial [30]. Nonspecialist HCPs working at intervention sites formed group 1 and received the comprehensive training, whereas those at control sites (group 2) received a shorter training.

### Participants

The training targeted 46 nonspecialist HCPs, including nurses and auxiliary nurse midwives (ANMs). One additional female participant serving in the role of psychosocial counselor also took part in the training and was included in the analysis.

Nurses and ANMs were selected because they are the primary providers of maternal and reproductive health services in Nepal and are often the first point of contact for women experiencing violence or mental health concerns. Furthermore, evidence suggests that women are more likely to disclose sensitive issues such as IPV to female care providers. Group 1 included 24 participants from intervention facilities, and group 2 included 22 participants from control sites.

### Ethical Considerations

This study forms part of a larger trial evaluating a multicomponent intervention to reduce IPV and psychological distress among women in Nepal [30]. The larger trial was registered with ClinicalTrials.gov (NCT05426863). Ethical approval was obtained from the Nepal Health Research Council (852/2019). All procedures adhered to the 2019 National Ethical Guidelines for Health Research in Nepal [31] and to the principles of the 1964 Helsinki Declaration and its subsequent amendments.

Written informed consent was obtained from all participating HCPs for both training participation and in-depth interviews. Consent included approval for audio recording and use of deidentified data. All data were stored securely on password-protected devices accessible only to the research team. Quantitative findings were reported in aggregated form to prevent participant identification. Audio files were deleted after transcription. Personal identifiers were removed from transcripts to maintain confidentiality. Verbatims used in this manuscript are labeled with nonidentifying labels.

HCPs received a daily allowance of either 1200 or 1600 Nepalese rupees for participation in the training, in accordance with the Nepalese government’s daily subsistence allowance rates, which vary based on the participant’s official ranking or position. Photographs of training sessions were taken only after obtaining written informed consent from participants, who explicitly indicated no objection to the use of images in study outputs. To protect privacy, faces of training participants shown in this paper were blurred.

### Training Duration

Training was conducted from May 26 to June 4, 2022, in Bardibas, Mahottari District, Madhesh Province. Nonspecialist HCPs in group 1 received a comprehensive 10-day training, whereas group 2 participants attended a 3-day training. Each training day comprised approximately 8 hours of instruction.

### Training Content

The training was grounded in the WHO’s Problem Management Plus framework [32], a brief psychological intervention designed for individuals experiencing distress in adversity-affected settings. Problem Management Plus has been demonstrated to be effective in LMIC contexts, including Nepal [33-35]. The intervention was further adapted to include modules on safety planning [36]. Details of the training content are provided in Textbox 1 . Nonspecialist HCPs at both sites were trained on different mental health–related topics, such as an introduction to mental health and common mental health problems, consequences of mental health problems on women, their family, and the community, and stigma associated with mental health problems. They were further trained on the role of nonspecialist HCPs in managing mental health problems and referring patients to higher health facilities, psychiatrists, or psychologists for further management. Nonspecialist HCPs were also trained in administering data collection tools. Furthermore, nonspecialist HCPs in group 1 were trained to identify patients with mental health problems, implement stress management techniques such as slow breathing exercises, apply problem identification and solving techniques, develop safety plans against IPV/DV, and manage challenging/difficult patients.

Textbox 1. Content of the training for DeVI intervention. *Integrated multicomponent intervention refers to the DeVI intervention comprising PM+, safety planning, and mental health components. ** Group 2 nonspecialist HCPs were informed about available social support services in the community but did not receive training on guiding survivors to enhance social support

Both groups (group 1 and group 2)

- Introduction to intimate partner violence (IPV)/domestic violence (DV): types of violence, causes and effects, and their impact on women, children, and society; and common health consequences of IPV/DV
- Global, national, and provincial epidemiology of IPV/DV and mental health problems
- Introduction to mental health and different mental health problems: causes, effects, and impact on women, children, and society; and stigma and mental health
- Relationship between IPV/DV and mental health
- Social support: approaches to strengthening and practicing support**
- Ethical guidelines on human research participants: the importance of consent, privacy, and confidentiality

Group 1 only

- Role of health care settings in addressing IPV in Nepal
- Detail on the integrated multicomponent intervention*, understanding adversity, and violence
- Use of basic helping skills to build relationship with participants
- Cyclical nature of violence and its phases
- Stress management: slow breathing techniques and deep breathing exercise
- Problem management: steps for managing problems, identifying solvable and unsolvable problems, and making problem management plans for solvable problems of the women experiencing violence
- Understanding “get going, keep doing” strategy to keep the women experiencing violence active and present
- Safety planning: developing safety plans for the participants for their protection from future harm and their advantages
- Assessing safety behaviors using checklists; exploring barriers to and facilitators of using safety behaviors
- Dealing with challenging clients
- Social support; and approaches to strengthening and practicing support, including steps of strengthening social support
- These contents were developed and moderated to the participants via different approaches such as discussion on real-life lived experiences by mental health professionals demonstrating treatment success stories.

- These concepts were not only delivered theoretically but were augmented through case examples based on our formative research in Madhesh province; interactive role-plays and reflection exercises were also added by the mental health professionals.

Nonspecialist HCPs were provided with brochures containing referral sites for women with mental health problems and a training manual, in line with the WHO’s recommendations for conducting intervention research on violence against women [37].

### Training Delivery

**Figure 1.**
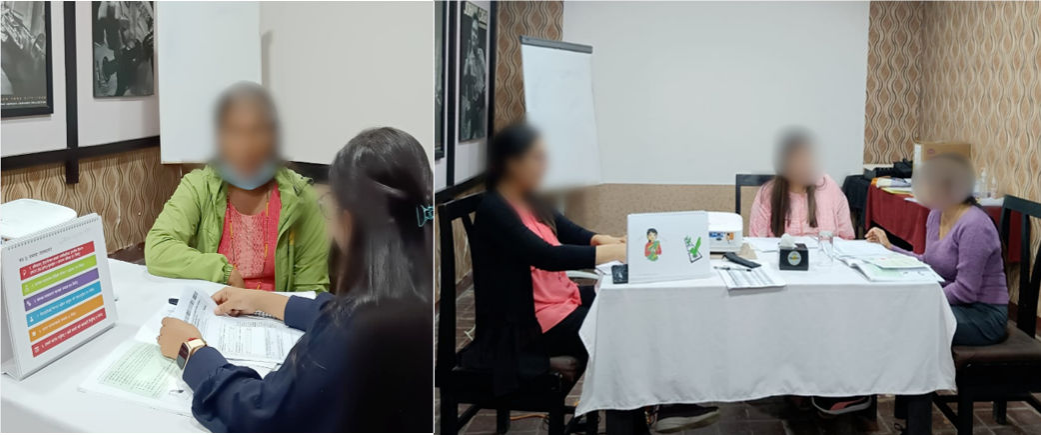
Glimpses of role-play activities during the training session. Left: Participants engaging in role-play as client and patient. Right: The training facilitator (psychiatrist) observing the role-play.

Training methods included didactic lectures, interactive group discussions, case vignettes, role plays, demonstrations, and brainstorming sessions (Figure 1). Activities were designed to maintain engagement and facilitate experiential learning. Recreational activities were incorporated to reduce fatigue during long training sessions.

Sessions were delivered in Nepali by a multidisciplinary team, including mental health professionals, public health experts, and members of the study team.

Participants demonstrated active engagement during training sessions. Role-play exercises using different scenarios were integrated into the training sessions (Figure 1). Experts demonstrated how interventions should be delivered. Nonspecialist HCPs also put forward different situations that could arise during the implementation of the intervention, which were addressed by experts via role plays. Role plays were evaluated by trainers based on predefined criteria, including confidentiality, nonjudgmental listening, and appropriate application of stress management and problem-solving techniques.

### Data Collection Tools and Variables

Quantitative data were collected using a self-administered questionnaire that included demographic information and 3 validated instruments.

Mental health knowledge schedule (MAKS) [38]: A 12-item tool scored on a 5-point Likert scale. The first 6 items in the scale measure the stigma-related mental health knowledge areas while the other 6 measure the knowledge on mental health problems. For positive statements, “strongly agree” is scored the highest 5 points whereas “strongly disagree” is scored 1. The points are reversed for the negative statements. Total scores are obtained by adding the scores of 12 items and higher score indicates higher participant’s knowledge in mental health.

Social Distance Scale (SDS) [39]: A 7-item scale assessing attitudes toward individuals with mental illness. Responses are rated on a 0 to 3 scale, with higher scores indicating a greater desire to distance one from the people with mental health problems. An example of an item is, “How would you feel about renting a room in your home to a person with serious mental illness?”

Perceived Dangerousness Scale [40]: An 8-item tool assessing participants’ beliefs about the dangerousness of individuals with mental health problems. Each item is scored from 0 to 5, with higher scores indicating greater perceived threat.

Although a more detailed version of SDS is available, we aimed at maintaining brevity while ensuring content validity for use in training evaluation context. All tools underwent cultural and linguistic adaptation following standard translation and back-translation procedures. A panel of Nepali mental health and public health experts reviewed the instruments for content and cultural appropriateness before finalization. It was then reviewed by a Nepali language expert to confirm cultural appropriateness and clarity. Lastly, the tools were finalized after consensus within the study team.

Qualitative data were collected using an interview guide focused on participants’ perceptions of the training, changes in knowledge, confidence, and self-reported skills. In-depth interviews were conducted only with group 1 participants due to the comprehensive nature of their training.

### Data Collection Techniques

The pretest, post-test, and follow-up questionnaires were self-administered questionnaires in Nepali language and took on average 15 to 20 minutes for completion. The pretest assessment was conducted prior to the training, while the post-test assessment was conducted after completion of the training. The follow-up questionnaire was completed after 3 months of training. In-depth interviews were conducted face-to-face and were audio-recorded with written consent from nonspecialist HCPs after completion of the training.

Quantitative data were self-administered by participants. In contrast, qualitative data were collected through interviews conducted by a mix of health workers, some of whom were involved in implementing the training sessions and others who were not. This approach was employed to capture detailed and diverse perspectives on participants’ experiences.

### Data Management and Analysis

#### Quantitative Analysis

Quantitative data were analyzed using STATA (version 14.2). Both descriptive and analytical analyses were performed. Sociodemographic characteristics are presented in the form of frequency, percentage, mean (SD), and median (IQR).

For MAKS, SDS, and Perceived Dangerousness Scale, data normality was assessed. Repeated-measures ANOVA was used for normally distributed data, and the Friedman test was used for nonnormally distributed data to compare mean scores at 3 different time points within groups [41]. Each item on MAKS was scored for each HCP. The scores obtained were then added to get the total score of each HCP. From the total score of each HCP, a mean score along with standard deviation was calculated. A similar process was conducted for the other two, perceived dangerousness of mental health patients and social distance scale as well. In both of these attitude scales, first, each item for individual HCP was calculated to get the total score obtained by them. As for assessing the effect of training between group 1 and group 2, one-way ANOVA for normally distributed data and the Kruskal-Wallis test for data not following normal distribution were performed [41]. The *P* value of ≤.05 was considered statistically significant.

#### Qualitative Analysis

Interviews were transcribed in Nepali and translated into English. Data were analyzed using thematic analysis [42]. Initial coding was based on concepts such as “first mental health training,” “stress management,” “confidence,” and “referral.” Themes were then refined through iterative discussion and mapped to the study objectives. Key themes included acquisition of new skills, changing perceptions, and increased readiness to provide psychosocial support.

The analysis focused on the mental health training component. Findings related to the IPV-focused elements of the DeVI will be presented separately due to differing timelines and follow-up periods.

## Results

### Participant Characteristics

Table 1 presents the sociodemographic characteristics of the 46 nonspecialist HCPs included in this study. The mean age of participants was 34.2 (SD 6.96) years, ranging from 21 to 50 years. Participants aged 21 to 30 years accounted for 39% of the sample (18/46), those aged 31 to 40 years also accounted for 39% (18/46), and participants aged 41 to 50 years constituted 22% (10/46). The majority of participants were married (38/46, 83%). More than half of participants were ANMs and senior ANMs combined (30/46, 65%), one-third were nurses (15/46, 33%), and 1 participant was a psychosocial counselor (1/46, 2%).

**Table 1.** Sociodemographic characteristics of the training participants (nonspecialist health care providers).

Variables	Group 1, n (%)		Group 2, n (%)		Total, N (%)
	Hospital	PHCC^a^	Hospital	PHCC^a^	
Age distribution (y)					
21‐30	6 (13)	2 (4)	1 (2)	9 (20)	18 (39)
31‐40	7 (15)	5 (11)	1 (2)	5 (11)	18 (39)
41‐50	3 (7)	1 (2)	0	6 (13)	10 (22)
Marital status					
Married	11 (24)	6 (13)	2 (4)	19 (41)	38 (83)
Unmarried	5 (11)	2 (4)	0	1 (2)	8 (17)
Profession within health facility					
ANM^b^	4 (9)	3 (7)	1 (2)	9 (20)	17 (37)
Nurse	8 (17)	4 (9)	1 (2)	2 (4)	15 (33)
Senior ANM	3 (7)	1 (2)	0	9 (20)	13 (28)
Psychosocial counselor	1 (2)	0	0	0	1 (2)
Level of education					
Proficiency certificate level in nursing	5 (11)	4 (9)	1 (2)	7 (15)	17 (37)
ANM	5 (11)	4 (9)	1 (2)	9 (20)	19 (41)
Bachelors	5 (11)	0	0	3 (7)	8 (17)
Master’s and above	1 (2)	0	0	1 (2)	2 (4)
Duration of working in current health facility (in y)					
<1	0	0	0	3 (7)	3 (7)
1-5	8 (17)	5 (11)	2 (4)	10 (22)	25 (54)
>5	8 (17)	3 (7)	0	7 (15)	18 (39)
Number of patients attended per week					
≤50	13 (28)	2 (4)	2 (4)	8 (17)	25 (54)
>50	3 (7)	6 (13)	0	12 (26)	21 (46)
Ever received any training related to mental health					
Yes	6 (13)	0	0	0	6 (13)
No	10 (25)	8 (20)	2 (4)	20 (50)	40 (87)

a PHCC: primary health care center.

b ANM: auxiliary nurse midwife.

Over half of the nonspecialist HCPs had worked at their current health facility for 1 to 5 years (25/46, 54%), and more than one-third over 5 years of service (18/46, 39%). Just over half of the participants reported attending to 50 or fewer patients per week (25/46, 54%).

Although nonspecialist HCPs often receive in-service training, the majority (40/46, 87%) reported that this was their first exposure to training focused on mental health. Only 6 out of 46 nonspecialist HCPs (13%) stated that they had earlier received training on mental health, particularly on psychosocial counseling. None of the participants from group 2 or from primary health care facilities in group 1 had previously received training on mental health (Table 1).

The in-depth interviews highlighted the importance of mental health–related training.

### Qualitative Findings From In-Depth Interviews

In-depth interviews conducted with group 1 participants revealed a range of positive changes in knowledge, confidence, and skills following the training.

### Enhanced Knowledge of Mental Health and Violence

Participants shared that the training enhanced their understanding of the interrelationship between mental health and IPV, equipping them to better identify, assess, and support affected individuals.

*This training definitely helped me. It is because with this training I have learnt how to manage women experiencing IPV... I can make safety plans for them which will help them in stress management.*

[Hospital Nursing Inspector, PHCC]

*Before this, we never considered managing confidentiality or asking them questions... Now we understand how to ensure privacy.*

[ANM, Hospital]

### Shift in Attitudes Toward Mental Health and IPV Survivors

Participants reported that the training changed their understanding of mental health. They earlier believed that mental health problems could only be solved via clinical interventions, but now they consider it a social issue that can be managed by counseling and empathy.

*We used to think that any mental illness needs medication. Now we know that it can be solved through counseling... and letting them express.*

[ANM, Hospital]

*Mental health problems were never prioritized. This training has made me realize it’s a key part of our work.*

[Staff Nurse, Hospital]

### Positive Training Experience and Perception of Training Modules

Participants valued the structured nature of the training, particularly the sessions on problem management and safety planning.

*Session on problem management was my favorite. We list solvable and unsolvable problems, develop a plan, and help women build routines. This helps them not feel isolated.*

[Hospital Nursing Inspector, PHCC]

*Training sessions helped identify the level of mental stress and how it could lead to suicide. These sessions were invaluable.*

[Nursing Officer, PHCC]

### Increased Confidence in Providing Psychosocial Support

Participants expressed that the training boosted their confidence in identifying and addressing mental health issues, even for those with no prior exposure.

*My confidence level has improved. I became aware of data collection, counseling steps, and follow-ups.*

[Staff Nurse, Hospital]

*Now I can identify mental health problems and provide support. This was missing from our service delivery before.*

[Staff Nurse, Hospital]

*Before this I have neither worked on anything related to mental health nor received any training. This is my first experience. But now we understand that, from where we work and the position we are in, we can assist individuals with mental health problems. We have also developed confidence that we can provide psychosocial counselling to them. Additionally, we learned about the organizations where we can refer patients with mental health problems.*

[Nurse, Hospital]

### Perceived Improvement in Counseling and Supportive Skills

Participants shared that the training enhanced their ability to manage mental health problems, particularly in stress management. Nonspecialist HCPs reported notable improvement in supportive skills like screening, problem identification, and active listening. Some also mentioned that they are now aware of referral sites for more advanced care.

*Earlier I did not know how to manage mental health problems. Through this training, we learned techniques like stress management, breathing exercises, and staying active. We also learned where to refer patients.*

[Nurse, PHCC]

*We learned relaxation techniques, deep breathing, and how to build rapport... Even a minor decrease in stress changes their daily life.*

[Staff Nurse, Hospital]

*Now we guide them to make their own decisions and plan for themselves. This is a skill I didn’t have before.*

[Nursing Officer, PHCC]

*We learned to identify mental health problems early, screen them properly, and maintain confidentiality.*

[ANM, Hospital]

### Changing Perceptions

Nonspecialist HCPs emphasized that mental health is often neglected in Nepal; this training helped to fill critical gaps in awareness, empathy, and basic support strategies. They recognized that simple interventions, such as counseling and stress management, could mitigate adverse outcomes, including depression or suicidal ideation. Overall, the training was perceived as essential in equipping nonspecialist HCPs with the necessary skills to help reduce mental health problems within their communities, marking a transformative change in their perceptions and approaches to mental health care.

*Mental health trainings are important and not common in Nepal. Most of us were unaware of how to interact with people with mental health problems, who are often stigmatized and isolated. This training showed us the impact of even small conversations and motivated us to engage with those showing signs of mental stress. We’ve learned we can help reduce mental health problems in our communities.*

[ANM, Hospital]

*This training has been beneficial. Before, we had limited knowledge and couldn’t effectively help patients with mental health problems, who are often discriminated against. Now, I feel confident in supporting them*.

[Nurse, Hospital]

*Minor issues can greatly affect mental health. If not addressed, stress can lead to depression or even suicide. By providing counseling and stress management, we can prevent these complications.*

[Nurse, Hospital]

### Quantitative Results

Table 2 summarizes changes in outcome measures before and after the training. In group 1, MAKS scores improved significantly from pretest to post-test (mean difference [MD] 7.41, 95% CI 4.85‐9.98) and remained elevated at 3-month follow-up (MD 7.08, 95% CI 4.28‐9.87). Group 2 also showed modest improvements at post-test (MD 3.82, 95% CI 0.01‐7.63) and at follow-up (MD 3.09, 95% CI 0.46‐5.71). Attitudes, as measured by the Perceived Dangerousness Scale, declined post-training in both groups (Table 2). Group 1 showed reductions at post-test (MD 2.30, 95% CI 0.27‐4.30) and 3 months follow-up (MD 4.67, 95% CI 2.33‐6.99). Group 2 also improved, with reductions at post-test (MD 4.77, 95% CI 2.60‐6.93) and 3 months follow-up (MD 6.91, 95% CI 4.17‐9.63).

SDS scores decreased in both groups post-training. Group 1 reported reduced scores at post-test (MD 1.87, 95% CI 0.02‐3.77) and follow-up (MD 2.87, 95% CI 0.75‐4.99). Group 2 demonstrated similar reductions at post-test (MD 3.50, 95% CI 0.82‐6.17) and follow-up (MD 2.45, 95% CI 0.11‐5.02) (Table 2).

**Table 2.** Comparison of knowledge and attitude within and between groups in pre, post, and 3 months follow-up.

Scales	Time	Group 1 (n=24), mean (SD)	*P* value^a^	Group 2 (n=22), mean (SD)	*P* value^a^	Total (n=46), mean (SD)	*P* value^a^	*P* value^b^
Mental health knowledge schedule	Pretest	41 (6)	<.001	41 (7.8)	.044	41 (6.7)	<.001	.94
	Post-test	49 (3.5)		45 (7.2)		47 (5.8)		.028
	Follow-up	48 (5)		44 (7.1)		46 (6.4)		.027
Perceived dangerousness of mental health patients	Pretest	20 (5.6)	<.001	19 (5.7)	<.001	19 (5.6)	<.001	.72
	Post-test	17 (6.9)		14 (4.9)		16 (6.2)		.092
	Follow-up	15 (5.9)		12 (3.2)		14 (4.9)		.051
Social Distance Scale	Pretest	9 (5.3)	.009	8 (4.9)	.011	8 (4-11)^c^	.001^c^	.85^d^
	Post-test	8 (4.9)		4 (3.3)		4 (2-8)^c^		.059^d^
	Follow-up	6 (4.5)		5 (2.6)		5 (3-8)^c^		.96^d^

a Within group *P* values analyzed using repeated measures ANOVA or Friedman test.

b Between group *P* values analyzed using one-way ANOVA or Kruskal-Wallis test.

c The total score for pre, post, and 3-month follow-up in the Social Distance Scale did not follow normal distribution; thus, the Friedman test was performed, and median scores with interquartile range were reported.

d For between analyses, *P* value was based on Kruskal-Wallis test.

### Between Group Comparisons

One-way ANOVA showed no significant pretest differences in MAKS scores between groups. However, post-test and follow-up scores were significantly higher in group 1, suggesting a stronger training effect. Similarly, for the Perceived Dangerousness Scale, significant between-group differences emerged only at the 3-month follow-up. Kruskal-Wallis tests for the SDS showed significant between-group differences post-training, but not at follow-up (Table 2).

## Discussion

### Principal Findings

This study found that nonspecialist HCPs had limited prior knowledge of mental health, as many were exposed to mental health–related training for the first time. This finding is consistent with previous studies indicating that individuals with mental health problems in LMICs often lack adequate care due to the limited mental health knowledge among HCPs [27,43]. Although Nepal has initiated mental health Gap Action Program-based mental health training programs through government and development partner collaborations [44], our earlier work showed that the majority of HCPs (almost 90%) had never received formal mental health training [12]. Consistent with previous research in Nepal [28] and international contexts such as Hong Kong and China [45,46], our study demonstrated an increase in mental health knowledge post-training in both groups. To help sustain knowledge gains, the study team conducted brief reviews of key training content during monthly regular monitoring visits.

In addition to improving knowledge, the training positively influenced HCPs’ attitudes [27]. While mental health issues are largely associated with IPV, they are often perceived as dangerous by both the public [47] and HCPs [48]. This perception was evident in our study during the pretest but showed a positive shift following the training.

Existing literature suggests that HCPs often hold stigmatizing beliefs and maintain social distance from individuals with mental health conditions [49,50]. At baseline, nonspecialist HCPs demonstrated moderate social distance scores. After training, both groups showed a reduction in these scores, reflecting improved attitudes toward people with mental health problems. Notably, group 1 maintained this reduction at the 3-month follow-up, whereas the effect was not sustained in group 2. This may be attributed to the more comprehensive and intensive training to the group 1 and prior exposure to mental health training or psychosocial counseling among some of its participants. While social distance scores differed significantly between the groups immediately post-training, the difference was no longer significant at follow-up, indicating that initial effects were not sustained and highlighting the need for ongoing support or refresher training.

Interestingly, group 2 exhibited greater improvement than group 1 in some attitude-related measures such as the SDS. This could be due to informal exposure to mental health concepts through personal experiences or ongoing discussions within their health facilities or communities. Previous literature has shown that even limited, contextually relevant exposure to mental health topics can influence stigma-related attitudes [51].

HCPs’ desire to distance themselves from individuals with mental health problems is influenced by their attitudes [52]. The training led to improved attitudes among nonspecialist HCPs, reducing their desire to distance themselves from those with mental health conditions. Prior to the intervention, many HCPs lacked the skills to manage mental health concerns or were unfamiliar with referral systems. The training addressed this gap, enhancing their perceived ability and self-reported readiness to identify symptoms, provide psychosocial support, demonstrate stress management techniques, and make appropriate referrals. For many, this was often their first structured training related to mental health, which helped foster a more supportive and responsive attitude toward affected individuals.

### Strengths and Limitations

A key strength of this study was the inclusion of a comparison group. To our knowledge, this is among the first studies in Madhesh Province to assess mental health knowledge and attitudes among nonspecialist HCPs at different levels of health care system. The study design captured both immediate and 3-month follow-up outcomes. The training employed interactive and practical methods, including role plays, case discussions, video demonstrations, and practice interviews, which align with established criteria for effective educational interventions [53]. The target population comprised nonspecialist HCPs at public health facilities, who are expected to apply the knowledge and skills gained from the training in their daily services, which is particularly beneficial given their long-term careers within the public health system. Moreover, the training model used in this study should be integrated into routine training within the health system, especially for health workers at district and primary health care levels, to address psychological distress and to facilitate appropriate referrals when necessary.

Nonetheless, the study has limitations. It did not assess whether changes in knowledge and attitudes led to behavioral changes in clinical practice. The small, all-female sample limits generalizability, although this is justified given the nurses and ANMs predominantly provide maternal and child health services in Nepal. The findings are therefore most relevant for similar provider groups. Moreover, the study did not adjust for potential confounders such as age or experience due to the small sample size (n=46), which may result in potential residual confounding. While no validated self-efficacy scale was used to measure confidence, this was addressed through qualitative data that provided insight into perceived changes in non-specialist HCPs’ confidence and practice.

### Future Implications

This training could be an important intervention for managing mild to moderate psychological distress and making appropriate referrals for severe mental health problems among women experiencing IPV or DV. In the present study, both groups received training; group 1 received comprehensive 10-day training while group 2 received short 3-day training. After the training, there was improvement in mental health knowledge and a positive change in attitude of nonspecialist HCPs across both groups, although the level of improvement varied between the groups. This suggests the possibility that brief training lasting for 3 days can bring some improvement among nonspecialist HCPs regarding their mental health knowledge and perception toward people with mental health problems.

### Conclusions

The structured training program significantly improved mental health knowledge among nonspecialist HCPs in Madhesh Province. At the end of the training, group 1 showed greater improvements post-training and at follow-up. Attitude toward mental health also shifted positively. Beyond improving knowledge and attitudes, the training helped equip HCPs with essential skills in counseling, stress management, problem-solving, and referrals. Such training is especially vital in settings with limited specialist services, where nonspecialist HCPs are often the first and only point of contact for individuals facing psychological distress and IPV.

## Abbreviations

ANM: auxiliary nurse midwives
DeVI: domestic violence intervention
DV: domestic violence
HCP: health care provider
IPV: intimate partner violence
LMIC: low- and middle-income countries
MAKS: mental health knowledge schedule
PHCC: primary health care center
RESHAPE: Reducing Stigma among Health Care Providers
SDS: Social Distance Scale
WHO: World Health Organization
